# Parental Effects on Epigenetic Programming in Gametes and Embryos of Dairy Cows

**DOI:** 10.3389/fgene.2020.557846

**Published:** 2020-10-14

**Authors:** Chongyang Wu, Marc-André Sirard

**Affiliations:** Centre de Recherche en Reproduction, Développement et Santé Intergénérationnelle (CRDSI), Département des Sciences Animales, Faculté des Sciences de l’Agriculture et de l’Alimentation, Université Laval, Québec City, QC, Canada

**Keywords:** parental age, embryonic development, dairy cows, epigenetics, metabolism, transgenerational inheritance, intergenerational inheritance

## Abstract

The bovine represents an important agriculture species and dairy breeds have experienced intense genetic selection over the last decades. The selection of breeders focused initially on milk production, but now includes feed efficiency, health, and fertility, although these traits show lower heritability. The non-genetic paternal and maternal effects on the next generation represent a new research topic that is part of epigenetics. The evidence for embryo programming from both parents is increasing. Both oocytes and spermatozoa carry methylation marks, histones modifications, small RNAs, and chromatin state variations. These epigenetic modifications may remain active in the early zygote and influence the embryonic period and beyond. In this paper, we review parental non-genetic effects retained in gametes on early embryo development of dairy cows, with emphasis on parental age (around puberty), the metabolism of the mother at the time of conception and *in vitro* culture (IVC) conditions. In our recent findings, transcriptomic signatures and DNA methylation patterns of blastocysts and gametes originating from various parental and IVC conditions revealed surprisingly similar results. Embryos from all these experiments displayed a metabolic signature that could be described as an “economy” mode where protein synthesis is reduced, mitochondria are considered less functional. In the absence of any significant phenotype, these results indicated a possible similar adaptation of the embryo to younger parental age, post-partum metabolic status and IVC conditions mediated by epigenetic factors.

## Epigenetics: A New Measure of the Effects of the Environment on the Phenotype

Phenotype is the term that refers to the observable characteristics or traits of an organism. It is determined by the genotype of an organism, and by the growing environment, but precisely predicting the actual outcome for a particular individual is still a challenge (Burga and Lehner, 2012). The idea that phenotype changes caused by environmental alterations can be passed to next generations has existed for centuries (Roberts and Hay, 2019) but only in recent decades the advances in epigenetics knowledge gave us insights to the underlying mechanism of how environment affects the phenotype of an organism. In this review, we will discuss the parental epigenetic effects on programming in gametes and embryos, mainly focusing on dairy cattle.

The definition of epigenetics is evolving to accommodate our increasing knowledge of mechanisms that regulate gene expression. Presently, the widely accepted definition of epigenetics is “heritable changes in gene expression without altering the DNA sequence” with two main characteristics: inheritable and reprogrammable (Allis and Jenuwein, 2016). Inheritability of epigenetics mainly refers to the maintenance of epigenetic modifications in a short term, such as during mitosis of differentiated somatic cells, while reprogramming of epigenetics describes the ability to erase and rebuild modifications in a long term, such as across generations (Ji and Khurana Hershey, 2012). However, reprogrammable epigenetic information does not mean it will be completely removed at each generation, on the contrary, a small portion of epigenetic modifications can be transferred to next generation (intergenerational inheritance) or even beyond them (transgenerational inheritance) (Perez and Lehner, 2019).

The understanding of epigenetics is constantly modulated by the advance in our knowledge of evolution and development (Felsenfeld, 2014). There are four main epigenetic components: DNA methylation, histone modifications, non-coding RNAs, and chromatin state. In contrast to genetic information, epigenetic factors are vulnerable to be influenced by environmental changes. These alterations have been reported to be transgenerationally and/or intergenerationally inherited and affect the phenotype of the subsequent generations (Xavier et al., 2019).

### DNA Methylation

DNA methylation is an epigenetic modification catalyzed by DNA methyltransferases (DNMTs) which transfer the methyl from the donor *s*-adenosyl methionine (SAM) to specific bases (Lyko, 2018). In mammals, DNA methylation predominantly occurs in the dinucleotide sequence 5′CpG3′, generating 5-methylcytosine (5mC) (Lyko, 2018). Most bulk genomic methylation status are steady over lifetime, with minor changes during specific cellular activities (Smith and Meissner, 2013). The two exceptions occur in embryo development, i.e., pre-implantation stage and primordial germ cell (PGC) stage, when most of the CpGs undergo global demethylation and remethylation (Wang et al., 2014). However, in mammals, a small portion of DNA methylation marks are protected from two waves of genome-wide DNA methylation reprogramming, which is not only indispensable for embryogenesis but can also inherit parental acquired traits to next generations (Smith et al., 2014; Wang et al., 2014; Jiang et al., 2018). The dynamic DNA methylome landscape carries important regulatory roles in gene expression, genomic imprinting, embryo development, and chromosome structure (Smith and Meissner, 2013).

### Histone Modifications

Histones are basic proteins in eukaryotic cell nuclei that package DNA into nucleosomes. Different covalent post-translational modifications of histones bind to different genomic elements and have diverse functions in modulating the chromatin structure or recruiting other proteins to regulate gene expression. Modifications such as histone H3 lysine 4 di-/tri-methylation (H3K4me2/me3), H3K36me3, H3K79me3, and histone acetylation are linked to active expression, while H3K9me2/me3, H3K27me3 and histone deacetylation are associated with transcriptional repression (Zhang et al., 2015). During spermatogenesis and maturation, histones are gradually replaced with protamine to reduce the size of the sperm head. However, 1–15% of the histones are retained and are associated with DNA regions accessible soon after fertilization (Siklenka et al., 2015). In contrast, this replacement does not occur in oocytes, but the histones go through spatial and temporal post-translational modifications, including acetylation, methylation, phosphorylation, etc., which is critical for oocyte maturation (Gu et al., 2010) and maternal-to-zygotic transition after fertilization (Dahl et al., 2016; Zheng et al., 2016; Xia et al., 2019). A novel imprinting mechanism was reported to be dependent on maternal H3K27me3 rather than DNA methylation (Inoue et al., 2017).

### Non-coding RNAs

Non-coding RNAs (ncRNAs) are transcripts that are not translated into proteins: such as ribosomal RNA (rRNA), ribozyme, transfer RNA (tRNA), and small nuclear RNA (snRNA). They are involved in the regulation of numerous bioactivities: such as cell proliferation, cell differentiation, cell apoptosis, cell metabolism, and chromosome remodeling. Moreover, ncRNA from both parental gametes are required for normal embryonic development (Yuan et al., 2016; Conine et al., 2018; McJunkin, 2018). Before the embryonic genome activation (EGA), early embryos mainly rely on transcript reservoir from maternal origin, thus the importance of maternal ncRNAs during early embryogenesis is taken for granted. Plenty of maternal ncRNAs were discovered to be involved in EGA, hence transfer maternal environmental influences to offspring (Perez and Lehner, 2019).

Sperm of germline-specific *Dicer* and *Drosha* conditional knockout mice have deficient miRNAs and/or endo-siRNAs profiles (Yuan et al., 2016). Although these sperms could still fertilize wild-type oocytes by intracytoplasmic sperm injection (ICSI), developmental potential of embryos produced were significantly impaired. Injecting wild-type sperm-derived total or small RNAs was able to rescue the developmental deficiency of these embryos (Yuan et al., 2016). Further studies identified that these transcripts especially small non-coding RNAs were gained during later maturation in epididymis (Conine et al., 2018). Embryos generated by ICSI using sperm from caput failed after implantation, while this deficiency could be rescued by microinjection of cauda-specific small RNAs (Conine et al., 2018).

### Chromatin State

Chromatin state is used to describe various conformations of chromatin structure which reflects its dynamic spatial changes during embryo reprogramming and other cellular activities (Zhou and Dean, 2015). Multiple epigenetic factors are involved in determining chromatin state, including histone modification, DNA methylation and non-coding RNAs (Gupta et al., 2010; Kundaje et al., 2015). The complex relationship between epigenetic modifications, chromatin state and transcriptional activities are still largely unknown, but our understanding of these associations is improving, based on the increasing experimental evidence (Ernst et al., 2011; Ernst and Kellis, 2012, 2017). Basically, genes with higher expression are most likely located at open chromatin regions which are generally modified by active epigenetic marks: such as H3K4me3 on proximal promoters, H3K27-ac on enhancers, and low DNA methylated promoters (Gaspar-Maia et al., 2011; Tian et al., 2011). Repressed expression genes are found in closed chromatin regions bound with inactive epigenetic marks including H3K37me3, hypermethylation at transcription start sites, and long non-coding RNA bound regions (Saxena and Carninci, 2011; Ando et al., 2019; Igolkina et al., 2019). During gametogenesis and embryogenesis, chromatin states undergo extensive remodeling to reach totipotency. However, the erasure is not complete, a complex pattern of 3D interactions between chromatin and transcription factors in both oocytes and sperm can still be transmitted to zygote according to ATAC-seq and ChIP-seq results (Jung et al., 2017, 2019). In bovine, retained histones were also identified by Mnase-seq in spermatozoa and seem required for spermatogenesis and fertilization (Sillaste et al., 2017). Overall, these retained accessible chromatin are indispensable for early embryogenesis and may also be potential candidates to transmit parental effects to next generations.

## Reprogramming and Inheritance of Epigenetic Marks During Embryo Development

### Preimplantation Embryo Development

Throughout mammalian life cycle, individual experiences two waves of global epigenetic reprogramming, during preimplantation stages and germ cell development (Zeng and Chen, 2019). After fertilization, murine parental genomes were both observed to be actively (TETs-dependent) and passively (replication-dependent) demethylated during early embryogenesis, with exception of certain genomic loci, including imprinted genes (Guo F. et al., 2014; Wang et al., 2014). Recent single-cell chromatin overall omic-scale landscape sequencing (scCOOL-seq) of mouse preimplantation embryos further comprehensively described a heterogeneous but highly coordinated features of epigenetic reprogramming (Guo et al., 2017). Global DNA demethylation occurs within 12 h of fertilization, while distinct gene regions were resistant to reprogramming in parental genomes (Guo et al., 2017). Comparable chromatin accessibility was observed between paternal and maternal genomes from the late zygote to the blastocyst stage (Wu et al., 2016; Guo et al., 2017). Genomic regions that are resistant to global epigenetic reprogramming are potential factors to inherit parental environmental influences on offspring, but this requires to be further distinguished from *de novo* modifications after fertilization.

Epigenetic reprogramming during early embryo development is conserved between human and mice, while the kinetics of human embryos is relatively slower (Eckersley-Maslin et al., 2018). In human, major wave of global DNA demethylation was completed at 2-cell stage, and further reduced to 29% as the bottom at blastocyst stage in ICM (Guo H. et al., 2014). Besides imprinted genes, evolutionarily young transposable elements with more active transcription retain their DNA methylation status in early embryos (Guo H. et al., 2014). scCOOL-seq of human preimplantation embryos identified a complex, yet highly ordered epigenetic reprogramming process (Li et al., 2018). In contrary to mice, the paternal genomes in human early embryos are already more open than maternal genomes from the mid-zygote to the 4-cell stage (Li et al., 2018).

In bovine, the genome-wide demethylation is closely related to EGA since the major wave reduction of DNA methylation is completed by the 8-cell stage (Jiang et al., 2018). During that period, promoter methylation is negatively correlated with the expression levels of genes at preimplantation stages; gametes-specific differentially methylated regions (DMRs) are enriched in different regions and demethylated in different manners (Jiang et al., 2018). However, a small portion of DNA methylation will be maintained during the global reprogramming after fertilization, including imprinted genes, which could become a legacy between parental environment effects with offspring phenotype (Jiang et al., 2018). Moreover, dynamic landscape of accessible chromatin in bovine preimplantation embryos were revealed recently by ATAC-seq. Chromatin accessibility is dramatically increased in coordination with EGA and reached to peak in elongating embryos at day 14 (Ming et al., 2020). Combined with bovine transcriptomic and DNA methylation data, accessible promoters were related to genes with high expression and the accessibility is closely related to DNA methylation level and CpG density (Ming et al., 2020). Although the expression of histone modification enzymes was profiled during early embryo development (McGraw et al., 2003; Glanzner et al., 2018), global reprogramming of modified histones throughout bovine preimplantation stages is not known.

### PGC Development

During the two waves, somatic epigenetic modifications in PGCs are erased and established as sex-specific patterns, including the genome-wide DNA methylation reprogramming and chromatin reorganization (Hackett et al., 2013). Less than 10% CpG sites were protected from demethylation in mouse PGC at E13.5, which is predominantly located in long terminal repeats (LTRs) at intergenic regions (Hackett et al., 2013; Wang et al., 2014). However, conflicting results were reported as to whether IAP elements (Intracisternal A-type particle) were resistant to DNA demethylation (Seisenberger et al., 2012; Hackett et al., 2013) or not (Wang et al., 2014). Along with the global DNA demethylation, chromatin structure undergoes actively remodeling by both extensive erasure of various histone modifications and exchange of histone variants (Hajkova et al., 2008). Although genome-wide distribution of retained histone modification is still not clear in mice, it has been identified that several silencing marks, such as H3K9me3 and H4K20me3, were retained on pericentric heterochromatin during PGC development (Magaraki et al., 2017). Diverse piRNAs were expressed in mice PGCs and involved in not only silencing of transposable elements but also translation regulations (Barreñada et al., 2020), which can be regarded as another transgenerational inheritance factors as reported in *Caenorhabditis elegans* (Ashe et al., 2012).

In human, the lowest DNA methylation was observed in the female PGCs of 10-week embryos, with an average of 6% methylation remaining (Guo et al., 2015). Similar to the observations in mice, the retained loci are particularly enriched in evolutionarily younger and more active repeat and transposable elements (Gkountela et al., 2015; Guo et al., 2015). Moreover, H3K9me3 can escape from global reprogramming to repress the constitutive heterochromatin (Gkountela et al., 2015; Guo et al., 2015), while the involvement of non-coding RNAs in human PGC development is still not clear.

Studies on PGC reprogramming were mainly focusing on model animals and human, although bovine PGCs were already identified and isolated by AP staining in embryos at E18-E39 in 1990s (Lavoir et al., 1994; Wrobel and Süß, 1998). Thus, it is urgently needed to characterize the landscapes of DNA methylation, histone modifications/chromatin states, ncRNAs, etc. during PGC development in bovine. This will enlighten our knowledge of the most extensively reprogramming process in large animals and point out the potential transgenerational inheritance factors which are able to escape from this global erasure.

## The Known Non-Genetic Parental Influences in Mice and Human

Due to the vast amount of information conveyed from female gametes to zygotes and the exposure to the uterus environment during pregnancy, epigenetic influences of maternal origin were studied extensively. The effects of maternal nutrition status, age, stress, lifestyle, disease, and others were reported to be transmitted to next generations. However, research on paternal non-genetic effects has been long neglected compared to the numerous studies undertaken on the maternal side. Benefitting from studies to identify the molecules that sperm transfer during fertilization, paternal epigenetic influences are now gaining more and more attention. Here, we will firstly illustrate parental non-genetic effects focusing on mouse and human studies.

### Non-genetic Maternal Effects in Mice

Due to the short lifespan, transgenerational studies of maternal effects in mice are quite common and informative. Pre-conceptional and gestational maternal obesity induced cardiac dysfunction and hypertension in offspring (Loche et al., 2018). Also, exposure to bisphenol A (BPA, an endocrine disruptor) induced metabolic defects transgenerationally up to the F3 offspring (Bansal et al., 2019). However, the phenotype was less severe with increasing generations, probably due to the diluting effects of epigenetic reprogramming during early embryo development (Bansal et al., 2019). Pre-conceptional maternal exposure to cyclophosphamide (an agent for breast cancer therapy) altered DNA methylation levels in F1 and F2 mouse oocytes, resulting in delayed growth in these two generations (Di Emidio et al., 2019).

### Non-genetic Maternal Effects in Human

In humans, the mother condition has a profound impact on offspring across generations through epigenetic modifications. Epidemiological studies demonstrated that F2 generations of mothers who experienced famine periods had a higher tendency toward metabolic disorders (Aiken et al., 2016), partially due to the alteration of methylation levels in genomic DNA and histones (Uchiyama et al., 2018; Zimmet et al., 2018). Maternal age is another factor that can influence the developmental outcomes of progeny. Offspring of younger mothers tended to take more time to get pregnant (Reynolds et al., 2020); while offspring of advanced-age mothers were more likely to have metabolism or neurodevelopmental disorders with reduced methylation levels of several specific CpG sites (Markunas et al., 2016). Hence, epigenetic modifications are involved in the massive non-genetic maternal influence on offspring.

### Non-genetic Paternal Effects in Mice

Several comprehensive studies on paternal epigenetic effects on offspring were generated with the mouse model. The effects of metabolic disorders in male mice (Wei et al., 2014) and toxic exposure (Guerrero-Bosagna et al., 2012) can be transgenerationally inherited by disturbing DNA methylation levels in sperm. Disruption of histone methylation during spermatogenesis resulted in modified chromatin states in sperm and impaired development and survivability over two generations (Siklenka et al., 2015). Transfer RNA-derived small RNAs (tsRNAs), the major component of ncRNAs in mature spermatozoa apparently acquired during the transit in the epididymis (Chen et al., 2016b; Sharma et al., 2018), is believed to mediate paternal diet-induced metabolic disorders in offspring (Chen et al., 2016a; Sharma et al., 2016). These results clearly demonstrated the transgenerational inheritability of paternal epigenetic information in mice.

### Non-genetic Paternal Effects in Human

In human, the paternal contribution to an embryo is required for the embryo development and non-genetic components are involved in the transmission of paternal acquired traits to their children. Alteration of DNA methylation level, histone modifications, non-coding RNA expression and chromatin status were observed in the sperm of men who smokes, are obese, or experience mental stresses indicating an intergenerational inheritance through them (Soubry et al., 2016; Jenkins et al., 2017; Rowold et al., 2017; Dupont et al., 2019). Moreover, some of these aberrant changes were found in the sperm of their offspring and transgenerationally influenced the health of next generation (Soubry et al., 2013; Craig et al., 2017).

## The Known Non-Genetic Parental Influences in Dairy Cattle

### Non-genetic Maternal Effects in Dairy Cattle

In bovine, prenatal maternal conditions were significantly correlated with the daughters’ fertility and milk production. For example, daughters of dam that calved early for the first time produced more first-lactation daily milk, had higher body condition score (BCS), but experienced difficulties conceiving (Banos et al., 2007). Meanwhile, gestating dams with higher BCS tended to give birth to calves with higher BCS, had lower return rates, but slightly lower daily milk yields (Banos et al., 2007). The prenatal environment of grand-dam can also somewhat influence the milk production in subsequent daughters potentially in a transgenerationally manner by epigenetic mechanisms (Singh et al., 2012; Gudex et al., 2014). Additionally, the separation of heifers from their mother shortly after birth, which is a widely applied procedure in dairy cow management, will result in stress analogous to the maternal separation and unpredictable maternal stress (MSUS) model in rodents. Thus, this practice makes dairy cows a model to study the non-genetic inheritance of MSUS (Engmann, 2018).

As mentioned above, the maternal metabolic status can influence production and reproductive traits of subsequent generations through non-genetic pathways. However, there is a paucity of studies on the specific mechanisms underlying the effects of the maternal metabolism pre-conception or during gestation on offspring. Recently, embryos from dairy cows experiencing different levels of negative energy balance (NEB) were collected to study the influence of the maternal metabolic environment on the transcriptome and epigenome of early embryos by microarrays (Chaput and Sirard, 2019). Transcriptomic data highlighted that the most significantly affected pathways were metabolism-related: such as protein synthesis (EIF2 Signaling and eIF4, translation factors), mitochondrial function (oxidative phosphorylation and mitochondrial dysfunction), and metabolism (Sirtuin signaling and mTOR Signaling) (Chaput and Sirard, 2019). Gene expression levels do not provide any information about the amount of protein generated, or the extent of further modifications: such as phosphorylation, acylation, and/or methylation. Transcriptomic studies can be used to evaluate the immediate impact of environmental stresses and the embryonic responses (Cagnone and Sirard, 2016). Meanwhile, to study the long-term effects of stressors, studies on epigenetic modifications are required, especially for those regions that are affected according to the transcriptomic results. Using a DNA methylation microarray, 462 DMRs were identified between embryos from cows in high and low NEB, with many of them being located in gene regions, including introns, exons, proximal promoters, promoters, and distal promoters (Chaput and Sirard, 2019). Most of these genic regions, except exons, were more hypermethylated in embryos from cows experiencing severe NEB (Chaput and Sirard, 2019). Functional analysis of DMRs located in gene regions was consistent with transcriptomic results and pointed toward metabolic related pathways (AMPK signaling and mTOR signaling) which are significantly affected by maternal energy deficits (Chaput and Sirard, 2019). As a key switch for keep the energy balance, AMPK were also regulated by small non-coding RNAs to control cellular anabolic and catabolic processes in dairy cows (Mahmoudi et al., 2015). These changes demonstrated the metabolic adaptations of embryos to the maternal gestational environment by the regulation of mitochondrial functions, cell growth, and other protective pathways. These NEB-associated DMRs were retained at the time of the global reprogramming after fertilization, thus it is highly possible that they could affect the phenotype of offspring intergenerationally. The DNA methylation analysis of blood from eight calves produced from high and low BHB mothers resulted in 1675 DMRs (*p* < 0.05) indicating a post-natal legacy. This study highlighted how embryos interact with the maternal environment and the potential for intergenerationally inherited phenotype transmission through epigenetic modifications.

### Non-genetic Paternal Effects in Dairy Cattle

Multiple epigenetic factors conveyed by sperm are affected by the environmental or physical conditions of bulls. Not only maternal and fetal effects influence gestation length, a total of 66,318 DMRs in sperm were correlated with gestation length as well as days to first breeding after calving, somatic cell score, body type, milk production, and other traits (Fang et al., 2019). Reactome pathways analysis further validated that DMRs were mainly related to pregnancy, embryonic development, and lipid metabolism pathways (Fang et al., 2019). Moreover, some of these DMR were mapped to genes that are transcriptionally active during preimplantation stages, suggesting their potential role in early embryo development (Fang et al., 2019). Similarly, age-related DMRs were observed in bovine spermatozoa (Takeda et al., 2017, 2019; Lambert et al., 2018), and 57 of 2223 DMRs (2.56%) were retained in blastocysts (Lambert et al., 2018; Wu et al., 2020). Among the genes that are mapped with these DMRs, some of them were involved in spermatogenesis (FKBP6) and embryonic preimplantation development (AKT2). Chromatin condensation status can also be altered under heat-stress and further influence DNA methylation reprogramming of paternal pronuclei, which may be responsible for the reduced fertilization rates after IVF (Rahman et al., 2014). *In vitro* exposure to Chlorpyrifos, a pesticide, significantly affected bovine sperm DNA methylation patterns, resulted in reduced sperm motility and IVF rates, and increased chromatin structure abnormalities (Pallotta et al., 2019). Although large epidemiologic studies are missing, these changes in the epigenetic marks could potentially alter the phenotype of offspring.

Similar to cows, paternal metabolic status significantly influences semen quality. Enhanced pre-pubertal nutrition elevates the percentage of progressively motile and upregulates mitochondrial function in sperm of post-pubertal dairy bulls (Johnson et al., 2020). In contrast, low planes of nutrition of young Holstein-Friesian bulls resulted in the retarded onset of puberty (Byrne et al., 2018). Although epigenetic studies related to bull metabolic status is still missing, DNA methylation and histone modifications patterns were both reported to be associated with bull fertility (Verma et al., 2015; Kropp et al., 2017; Kutchy et al., 2018; Capra et al., 2019; Ugur et al., 2019). Functional annotation of these alterations indicate that they might be involved in spermatogenesis and embryo development (Verma et al., 2015; Ugur et al., 2019).

### Effects of *in vitro* Culture Condition in Dairy Cattle

Another tool to study embryo programming is *in vitro* culture (IVC) in different types of environment. Assisted reproductive technologies (ARTs) have been widely used to either overcome reproductive difficulties (for human) or increase the genetic gain of elite sires (for cattle). *In vitro* culture, an indispensable ART procedure, allows zygotes to divide to a transferrable stage at around day 7 for bovine embryos. Each aspect of the IVC medium, including physicochemical, oxidative, and energetic conditions (Summers and Biggers, 2003), has profound effects on embryo development, and these effects could be maintained to adulthood or even subsequent generations. The physiochemical parameters increased osmolality (Etienne and Martin, 1979), decreased local pH (Dagilgan et al., 2015), and heat shock (Sakatani et al., 2008) were all shown to compromise embryo development. Additionally, specific levels of reactive oxygen species (ROS) are required for normal embryo development; however, it is inevitable that embryos will be exposed to higher concentrations of oxygen *in vitro*, which is detrimental as a result of increased H_2_O_2_ production, DNA fragmentation, and mitochondrial dysfunction-induced apoptosis (Van Soom et al., 2002; Harvey et al., 2004; Kitagawa et al., 2004). The presence of nutrients in concentrations that mimic *in vivo* conditions is fundamental to early embryogenesis as demonstrated by the deleterious effects on embryo development of high concentrations of glucose and lipids in the culture medium (Díaz-Ruiz et al., 2008). An excessive inflammatory response was observed in IVC blastocysts, and this could interfere with the embryo-maternal recognition process following transfer (Cagnone and Sirard, 2014). Based on the metabolic pathways affected by culture conditions, embryos can either enhance a Warburg-like effect to adapt to minor stresses or induce apoptosis under severe stress. As a result, even though blastocyst rates may not be significantly impacted in modified media, embryo loss rates may be higher compared to control conditions. Moreover, mitochondrial dysfunction is involved in the embryonic response to IVC stresses which may impact its main role as energy factory, as well as the production of acetyl-CoA and methyl groups associated with one-carbon metabolism (Steegers-Theunissen et al., 2013), which controls histone acetylation and DNA methylation. Thus, suboptimal IVC conditions do disturb the embryonic epigenome which could contribute to the sometime altered phenotype of offspring (Cagnone and Sirard, 2016).

## Using Parental Age to Assess the Mechanisms of Metabolic Programming

Potential genetically elite sires can be identified a few days postnatally by genomic selection. In combination with ARTs, these approaches greatly reduce generation interval to increase the rate of genetic gain (Kasinathan et al., 2015). Additionally, there is increased demand for the use of juvenile calves or heifers for embryo production in combination with genomic selection and ARTs, which can further shorten generation interval (Landry et al., 2016). However, gametes from young donors are suboptimum compared to those from adults. Recent studies also indicated the potential parental age effects on early embryo development.

### Paternal Age Effects on Semen

On the male side, semen quality and quantity are significantly correlated with the age of the bull (Takeda et al., 2017). The ontogeny of the male reproductive system is initiated during the fetal period and the onset of bull puberty is governed by complex neuroendocrine networks (Plant, 2015) which are responsible for the tight coupling between metabolic status and reproductive system development (Roa et al., 2010). The early transient rise in LH pulsatility marks the initiation of puberty (Evans et al., 1995), which is observed in bull calves between 10 and 20 weeks of age (Rawlings and Evans, 1995). Leydig cells proliferate rapidly with an elevated responsiveness to LH, resulting in increased testosterone concentration to initiate the differentiation of unmatured Sertoli cells and further spermatogenesis (Amann and Walker, 1983). The first unmatured spermatids are generated between 25 and 35 weeks of age, while mature spermatozoa are obvious in seminiferous tubules at 32–40 weeks of age (Abdel-Raouf, 1960; Macmillan and Hafs, 1968; Evans et al., 1996; Bagu et al., 2006). A bull is then considered to have reached puberty when the first ejaculation containing over 50 million spermatozoa with at least 10% progressively motile spermatozoa is observed at around 45 weeks of age (Wolf et al., 1965). Hence, it is expected that semen collected at prepubertal stages is often sub-optimal in terms of sperm concentration, motility, and IVF performance compared to semen collected from adult bulls (Takeda et al., 2017). Moreover, this sub-standard sperm quality is associated with lower body weight in sexually immature bulls (Devkota et al., 2008), indicating that the paternal age effects on reproductive outcome may resemble the restricted diet effects (Brito et al., 2007).

Several studies demonstrated that enhanced early-life nutrition of bull calves positively affects several key metabolic and reproductive hormones related to the hypothalamic-pituitary-gonadal (HPG) axis, as reviewed by Kenny and Byrne (2018), and successfully hastens puberty without interfering with post-pubertal semen quality (Dance et al., 2015, 2016). In the commercial environment (optimized for breeders) setting used for the bulls in our study, semen ejaculated at the age of 10 months performed similarly to semen from post-pubertal animals regarding sperm concentration, motility, and blastocyst rates; although the semen from younger animals contained fewer spermatozoa (Wu et al., 2020).

To study the epigenetic programming of sperm from young bulls, a new bovine specific platform, EmbryoGENE^1^, which can simultaneously evaluate the genome-wide epigenome and transcriptome of small samples, such as sperm, oocytes, and early embryos (Shojaei Saadi et al., 2014), was used to evaluate the influence of environmental and parental effects on embryo development (Salilew-Wondim et al., 2015; Desmet et al., 2016; Pagé-Larivière et al., 2016; Morin-Doré et al., 2017; Tremblay et al., 2018; Chaput and Sirard, 2019; Wu et al., 2020).

Different DNA methylation patterns were observed in sperm collected either from the same bulls at different pubertal stages (Lambert et al., 2018) or from different bulls of different ages (Takeda et al., 2017, 2019). Approximately 69% of the DMRs were located in genic regions, including one associated with the paternally imprinted gene MEST (mesoderm specific transcript) (Lambert et al., 2018). Methylation levels of most DMRs were higher with increasing age (Lambert et al., 2018; Takeda et al., 2019); interestingly, these levels changed rapidly especially at younger ages (Takeda et al., 2019). Network analysis of these DMRs revealed that sperm function related pathways, such as PKA signaling, sperm motility, calcium signaling, and protein G signaling pathways were significantly affected by paternal age (Lambert et al., 2018). Hence, although young bulls can produce functional semen, epigenetic factors transmitted by spermatozoa could potentially impact embryo or offspring development.

### Paternal Age Effects on Embryos

Epigenetic modifications are known to occur as paternal inter or transgenerational inheritance factors (Rando, 2016; Spadafora, 2017; Bošković and Rando, 2018). To further study paternal age effects on embryos, blastocysts were produced by IVF with spermatozoa from the same bulls at different pubertal periods (10, 12, and 16 months) and oocytes from several matched (the same cow for each individual bull) adult cows (Wu et al., 2020). Transcriptomic and epigenetic analysis were performed on four pairs, where the only difference between embryos was the age of the bull. The results revealed elevated mitochondrial dysfunction, suppressed oxidative phosphorylation, and reduced protein synthesis in blastocysts generated from younger bulls suggesting a low energy status in these blastocysts (Wu et al., 2020). Moreover, the affected metabolic and sperm function pathways observed in blastocysts were consistent with the sperm studies mentioned above (Lambert et al., 2018), suggesting paternal age effects on embryos mediated by epigenetic factors in sperm.

### Maternal Age Effects on Oocyte

On the female side, the same selection pressure pushes the breeding industry to collect oocytes from dairy heifers to reduce generational interval and increase genetic gain. It has been reported that *in vitro* produced embryos can be obtained using oocytes from heifers as young as 2–4 months; however, few cumulus oocyte complexes (COCs) could be recovered from non-stimulated heifer ovaries (Majerus et al., 1999; Palma et al., 2001; Kauffold et al., 2005). Even though these COCs had similar performance in maturation, fertilization, and early cleavage rates after IVF compared to COCs from adult cows, reduced blastocyst yields and greater embryo loss following embryo transfer were observed, which may be a consequence of increased apoptosis in embryos from young donors (Zaraza et al., 2010). Ovarian stimulation is widely accepted for generating oocytes of high quality and for increasing blastocyst yields from adult cows (Nivet et al., 2012; Labrecque et al., 2013). More follicles were aspirated from young calves following stimulation, and higher numbers of mature oocytes and cleaved embryos were obtained (Landry et al., 2016). However, there was no difference in the total number of morula or viable embryos from heifers 5–18 months old, due to the significant lower morula and blastocyst rates in heifer groups which neutralized the larger number of oocytes recovered (Landry et al., 2016). Further analysis of granulosa cells collected from heifers demonstrated that cell differentiation, inflammation, and apoptosis pathways were inhibited indicating a suboptimal environment for oocytes in young donors (Landry et al., 2018).

### Maternal Age Effects on Embryos

To further investigate the maternal age effects on embryos, oocytes from the same heifers at different pubertal stages were collected and *in vitro* fertilized with spermatozoa from matched adult bulls (Morin-Doré et al., 2017). Transcriptomic analysis revealed that mitochondrial function was impacted in blastocysts from younger heifers, with inhibited mTOR, NRF2, and PPAR signaling (Morin-Doré et al., 2017). Thus, we can obtain more oocytes and comparable numbers of transferable embryos from young donors following ovarian stimulation; however, young maternal age impairs metabolic functions during early embryo development and may cause embryo loss at later stages or induce offspring health disorders. The identification of genes affected in blastocysts from younger females revealed several gene pathways similarly affected in embryos originating from younger males making the results even more convincing and suggesting that there might be an evolutionary conserved mechanism involved.

## Genotype × Environment Interaction

**FIGURE 1:**
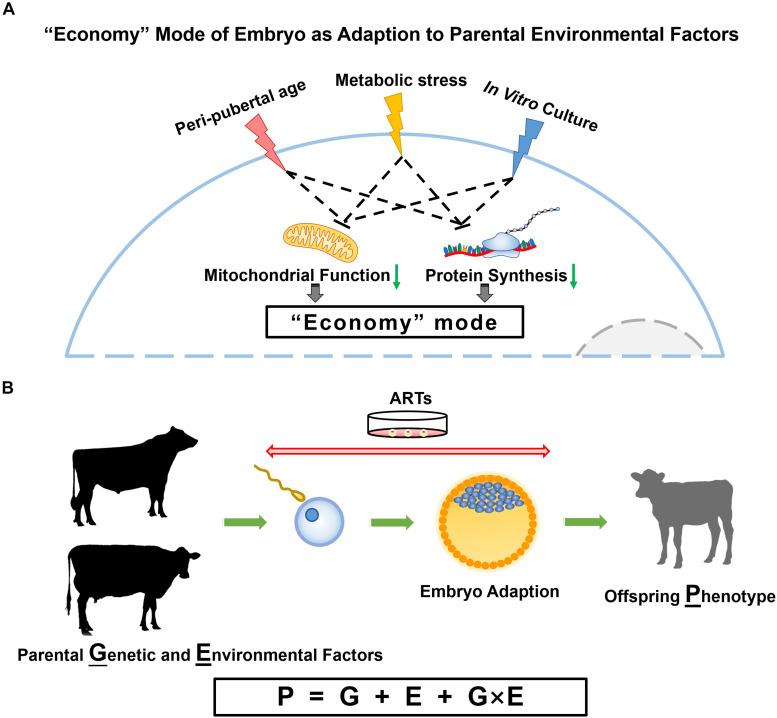
Parental influences on embryo and offspring. **(A)** Proposed model for embryo adaption of parental environmental factors. In this model, peri-pubertal age, metabolic status and IVC conditions result in embryos turning to an “economy” mode with slowed down cellular activities, especially the reduced mitochondrial function and protein synthesis. **(B)** The phenotype of offspring is determined by genotype, environment, and the interplay of genetic and environmental factors.

Genotype (G) × Environment (E) interaction is defined as genotype-specific phenotypic responses to different environments (Falconer, 1952). Taking G × E into consideration in dairy cow breeding reduces the error variance and hence increases the accuracy of genetic evaluation of sires and cows compared to classical approach, which considers the effects of genotype and environment only (Figure 1B) (Ron and Hillel, 1983). Two formations of G × E interactions can be taken to induce either unchanged ranking, i.e., a scaling effects, or reranking of sires across environments (Cromie et al., 1998). Plenty of across countries and within a country dairy cattle studies concluded that scale effects, which is due to the unequal scale of differences in sire proofs in the two environments, account majorly for the G × E interaction (McDaniel and Corley, 1967; Stanton et al., 1991; Boettcher et al., 2003). However, emerging evidence using genetic correlation proved the existence of re-ranking effects of G × E interactions, especially between the traits expressed in environments with large differences as reviewed by Hammami et al. (2009b) and Wakchaure et al. (2016). Genetic correlations < 0.80 were observed between milk yield across countries (Ojango and Pollott, 2002; Cerón-Muñoz et al., 2004b; Hammami et al., 2009a), between age at first calving across countries (Cerón-Muñoz et al., 2004a), between two feeding systems within country (Ramírez-Valverde et al., 2010), indicating the presence of genotype by environment interactions and at least some re-ranking of the animals. In this case, the breeders are required to optimize the breeding programs to accommodate the various environments.

Genes may be expressed in different patterns under different environment, and this could be one of the molecular mechanisms of G × E interactions (Hammami et al., 2009b). Epigenetic factors are involved in changing gene expression under varied environments. For example, DNA methylation at promoter regions can affect the binding of transcription factors, small non-coding RNAs can also regulate the transcription and translation in different ways, histone modification and chromatin states can determine the accessibility of chromatin for gene expression. Epigenetic regulation of multiple traits of dairy cow under different environments have been studied broadly and deeply (Singh et al., 2010; Ibeagha-Awemu and Zhao, 2015; Thompson et al., 2020). However, direct study of involvement of epigenetic factors in G × E interactions is still missing. Nevertheless, genomic bias were presented in relation to gene with G × E interactions, as reviewed by Grishkevich and Yanai (2013) based on whole-genome approaches in model organism and concluded that gene having long promoters with high concentration of regulatory motifs showed high correlation with distant-acting loci. Thus, epigenetic variations in different environments are highly possible to be responsible for G × E interactions effects, while substantial and systematic studies are required.

## Conclusion

Epigenetic information conveyed by gametes represent non-genetic factors that may explain why gametes of donors at different ages have similar reproductive performance, but result in different gene expression and DNA methylation patterns in embryos they produced. Counterintuitively, parental nutritional status, age, and IVC environment have similar consequences on embryo programming, i.e., alterations in metabolic pathways, especially mitochondrial signaling, which proves that cellular energy production is central in the response to environmental changes (Figure 1A). The most important task now becomes the analysis of post-natal phenotypes to identify the phenotypical consequences of all these epigenetic modifications in offspring. Fortunately, in sub-species like the dairy cow, data are accumulating on the genetic side (G) as more and more animals get genotyped, and on the phenotype (P) side using manual or electronic data generation at the farm. The combination of information from the farm environment (E) with genotype (G) and phenotype (P) information will allow the modeling of optimal environmental conditions for each animal or the optimal genotype for a defined environment (Figure 1B).

## Author Contributions

M-AS designed the manuscript. CW and M-AS wrote the manuscript. All authors read and approved the final manuscript.

## Conflict of Interest

The authors declare that the research was conducted in the absence of any commercial or financial relationships that could be construed as a potential conflict of interest.

## Footnotes

1 http://emb-bioinfo.fsaa.ulaval.ca/
